# Copper nanoparticles and silver nanoparticles impair lymphangiogenesis in zebrafish

**DOI:** 10.1186/s12964-023-01403-x

**Published:** 2024-01-25

**Authors:** YuanYuan Jing, ZhiPeng Tai, Jing-Xia Liu

**Affiliations:** College of Fisheries, Key Laboratory of Freshwater Animal Breeding, Ministry of Agriculture, Huazhong Agricultural University, Wuhan, 430070 China

**Keywords:** CuNPs, AgNPs, Lymphatic vessels, *CCBE1*, Hypermethylation, E2F7/8, ROS

## Abstract

Lymphatic system distributes in almost all vertebrate tissues and organs, and plays important roles in the regulation of body fluid balance, lipid absorption and immune monitoring. Although CuNPs or AgNPs accumulation has been reported to be closely associated with delayed hatching and motor dysfunction in zebrafish embryos, their biological effects on lymphangiogenesis remain unknown. In this study, thoracic duct was observed to be partially absent in both CuNPs and AgNPs stressed zebrafish larvae. Specifically, CuNPs stress induced hypermethylation of E2F7/8 binding sites on *CCBE1* promoters *via* their producing ROS, thereby leading to the reduction of binding enrichment of E2F7/8 on *CCBE1* promoter and its subsequently reduced expression, then resulting in defective lymphatic vessel formation. Differently, AgNPs stress induced down-regulated *CCBE1* expression *via* down-regulating mRNA and protein levels of E2F7/8 transcription factors, thereby resulting in defective lymphatic vessel formation. This study may be the first to demonstrate that CuNPs and AgNPs damaged lymphangiogenesis during zebrafish embryogenesis, mechanistically, CuNPs epigenetically regulated the expression of lymphangiogenesis regulator *CCBE1 via* hypermethylating its promoter binding sites of E2F7/8, while AgNPs *via* regulating E2F7/8 expression. Meanwhile, overexpression of *ccbe1* mRNA effectively rescued the lymphangiogenesis defects in both AgNPs and CuNPs stressed larvae, while overexpression of *e2f7/8* mRNA effectively rescued the lymphangiogenesis defects in AgNPs rather than CuNPs stressed larvae. The results in this study will shed some light on the safety assessment of nanomaterials applied in medicine and on the ecological security assessments of nanomaterials.

Video Abstract

**Supplementary Information:**

The online version contains supplementary material available at 10.1186/s12964-023-01403-x.

## Background

Nanomaterials are widely used in imaging, diagnosis, tissue engineering, surface texture, as detergents and even agriculture because of their antibacterial properties, unique mechanical properties, good plasticity and high hardness [1]. However, with the wide use of nanomaterials and their accumulation in environment, their harms to aquatic organisms even human beings have to be worried. Meanwhile, their widely use in medicine also raise the concerns of their safety in human beings. Therefore, it is important to study the biological effects of nanomaterials in organisms and to assess their safety in mammalian cells.

Copper nanoparticles (CuNPs) and silver nanoparticles (AgNPs) are two of the most widely used nanomaterials in commercial products and biomedicine due to their broad-spectrum antibacterial properties. CuNPs stress has been reported to induce renal dysfunction in mouse by increasing reactive oxygen and nitrogen species [2, 3], and damage the liver, kidney and spleen by causing mitochondrial stress in rats [4]. Additionally, CuNPs stress inhibits fish survival and growth and overfill blood vessels in a dose-dependent manner [5–7]. Meanwhile, studies have demonstrated that AgNPs stress induces neurodevelopmental abnormalities, cardiac development defects, erythrocyte and pigment production defects, and lens development defects in early embryonic zebrafish [8–11]. AgNPs stress also disrupts zebrafish skeletal and cardiac myofibrillogenesis and sarcomere formation [12]. Furthermore, AgNPs stress disrupts the function of fish’s olfactory [13]. Previous studies have shown that the promoter of gene *ccbe1* is hypermethylated and the gene exhibits down-regulated expression in copper (Cu^2+^) and AgNPs stressed embryos in zebrafish [7], and that Cu^2+^ stress impairs lymphangiogenesis by epigenetically regulating E2F7/8 transcriptional activity on gene *CCBE1* [14]. However, few studies have addressed the biological effects of CuNPs and AgNPs on development of lymphatic vessels in vertebrates and the underlying regulating mechanisms.

As parts of the vertebrate circulatory system, lymphatic vessels play important roles in maintaining fluid balance, intestinal fat absorption, and immune response [15, 16]. Abnormal development of lymphatic vessels often leads to lymphedema, inflammation, and even tumor. Lymphatic vessels have also been found to play roles in obesity, cardiovascular disease, and neurological disorders [17, 18]. Zebrafish has a short cycle of lymphangiogenesis and lymphatic vessel development, which is an excellent model for directly observing the embryonic and developing lymphatic vessel and studying the underlying molecular mechanisms. In zebrafish, budding of lymphatic vessels begin at 32 h post fertilization (hpf) [19, 20], and parachordal lymphangioblasts (PLs) are produced when somatic cells migrate from the dorsal side of the main vein to the horizontal septa for colonization [20–22]. These PLs then migrate along the arteries, both dorsal and ventral (starting at about 60 hpf), then, the main lymphatic vessels, thoracic ducts, intersegmental lymphatic vessels, and dorsal longitudinal lymphatic vessels are constructed [15, 23, 24]. Signaling factors, such as *ccbe1*, *flt4*, *vegfc*, *adamts3/14*, etc., play important regulatory roles in zebrafish lymphangiogenesis [25–28].

In this study, we unveiled that CuNPs and AgNPs stress impaired lymphangiogenesis during zebrafish embryogenesis. Briefly, we observed that some thoracic ducts (*fli1a*GFP^+^ *flk1*mCherry^−^) were absent in both CuNPs and AgNPs stressed Tg (*fli1a*:GFP; *flk1*:mCherry) embryos and larvae. Zebrafish embryos and human umbilical vein endothelial cells (HUVECs) stressed with either CuNPs or AgNPs exhibited down-regulated expression of *CCBE1*. Mechanistically, CuNPs stress induced ROS in zebrafish embryos and HUVECs, which might lead to the hypermethylation of the E2F7/8 binding site on *CCBE1* promoter and the significant reduction of their binding enrichments on the promoter, thus inducing the reduced expression of *CCBE1* and the resulted in lymphatic vessel defects. AgNPs stress down-regulated mRNA and protein level of E2F7/8 transcription factor in embryos and HUVECs, thus inducing lymphatic vessel formation defects. In addition, overexpression of *ccbe1* mRNA effectively rescued the lymphangiogenesis defects in both AgNPs and CuNPs stressed larvae, while overexpression of *e2f7/8* mRNA effectively rescued the lymphangiogenesis defects in AgNPs rather than CuNPs stressed larvae. This study revealed for the first time that the different regulatory mechanisms of CuNPs and AgNPs stress in impairing lymphangiogenesis by down-regulating *CCBE1*, which provides a theoretical basis for assessing safety of nanomaterials in medicine and in ecological environment.

## Materials and methods

The full names and abbreviations of the genes tested in this study are listed in Supplementary Table 1.

### Zebrafish maintenance and cell culture

Adult zebrafish (*Danio rerio*) were maintained in a circulating filtration system at 28.5 °C on a 14 h light/10 h dark cycle. Embryos were collected from the natural mating of breeding pairs (AB, Tg(*fli1a*:GFP; *flk1*:mCherry)) and raised at 28.5 °C, and the samples were collected at the indicated stages for experiments. HUVECs cells were grown in RPMI-1640 supplemented with 10% fetal bovine serum (FBS) in a 37 °C incubator containing 5% CO_2_. Stock solution of CuNPs (Cat#774,111, Sigma-Aldrich, U.S.A.) and AgNPs (Cat#730,807, Sigma-Aldrich, U.S.A.) were prepared with a fixed concentration respectively, then the stock solution separately was added to the culture medium to 0.15 mg/L and 0.40 mg/L for zebrafish embryos as we performed previously [5, 8], and to 0.75 mg/L and 2.0 mg/L, respectively, for HUVECs in this study. The concentration of antioxidant N-Acetyl-L-cysteine (NAC, Cat#8460, Solarbio, China) used in cultured medium was 200 µM, and NAC were added 1 h before CuNPs and AgNPs were added in the medium [5].

### Microscopy observation

The embryonic lymphatic structures from the control, CuNPs or AgNPs stressed groups; control, CuNPs or AgNPs stressed groups injected with *ccbe1* mRNA; control, CuNPs or AgNPs stressed groups injected with *e2f7/8* mRNA, respectively, at 120 hpf, were observed and photographed under a confocal microscope (Olympus FV1000 Confocal Microscope, Japan), to examine the lymphatic morphology using Tg(*fli1a*:GFP; *flk1*:mCherry) embryos and larvae, in which *fli1a*GFP positive while *flk1*mCherry negative cells are lymphatic vessel cells.

### RNA preparation and real‑time PCR analysis

Real‑time PCR (RT-PCR) was used to detect the expression of the candidate genes. In this study, the embryos and HUVECs of the control group, CuNPs stressed group, AgNPs stressed group and the corresponding reactive oxygen species [19] scavenger NAC treated group were tested separately in the transcriptional expression of genes *bip* (*heat shock protein 5*), *ire1a* (*tumor protein p53*), *perk* (*eukaryotic translation initiation factor 2 alpha kinase 3*), *flt4* (*fms-related receptor tyrosine kinase 4*), *vegfc* (*vascular endothelial growth factor C*), *ccbe1* (*collagen and calcium-bind-ing EGF domains 1*), *e2f7* (*E2F transcription factor 7*), and *e2f8* (*E2F transcription factor 8*). Total RNA was isolated from 32, 52 or 120 hpf zebrafish embryos or larvae using TRIzol reagent (Invitrogen). Oligo-dT-primed cDNA was synthesized using an M-MLV Reverse-Transcript Kit (Applied Biological Materials Inc, BC, Canada). Real-time PCR was performed using iQ™ SYBR® Green Super mix (Bio-Rad Laboratories, Hercules, CA, USA) in a CFX Connect™ Real-Time PCR Detection System (Bio-Rad Laboratories, Hercules, CA, USA). The specificity of all primers was tested before use, and the sequences of these primers are listed in Supplementary Table 2. Differences were calculated according to the 2^−ΔΔCt^ comparative quantization method using *18 s* as an internal control with one way analysis of variance [20] and post hoc Tukey’s test.

### Intracellular reactive oxygen species assays

In this study, ROS levels in embryos and HUVECs in control, CuNPs stressed, AgNPs stressed and the corresponding NAC treated groups were measured using A DCFH-DA (20,70-Dichlorodihydrofluorescein-diacetate) Reactive Oxygen Species Assay Kit (Beyotime, China), performed as manufacturer’s instructions [29, 30]. Then, the DCFH-DA-labeled embryos were photographed under a Stereoscopic Microscope (Leica M205FA). The DCFH-DA-labeled HUVECs cells were analyzed using CytoFLEX Flow Cytometer (Beckman Coulter, USA). At least 2–3 biological replicates were performed in this assay. All procedures were carried out in the dark [31].

### Whole‑mount in situ hybridization (WISH)

WISH was performed as described in our previous studies [6, 32]. Based on available information, gene specific primers were designed and shown in Supplementary Table 3. The PCR products were cloned into pGEM-T Easy vectors for anti-sense RNA probe synthesis using RNA labelling mix (Cat#AM1312, Thermo Fisher, USA). Then, the anti-sense RNA probes labeled with digoxigenin were used to test the expression of the target genes in different whole embryos by WISH analysis (Roche Diagnostics). The images were taken under a Leica microscope (Leica M205FA, Germany).

### mRNA synthesis and injection

The full-length of zebrafish genes *e2f7/8* and *ccbe1* were amplified using the primers shown in Supplementary Table 4. For mRNA preparation, capped mRNAs were synthesized using the mMessage mMachine kit (Cat#AM1344, Ambion, USA) according to the manufacturer’s instructions. The synthesized mRNAs were diluted into different concentrations and injected into one-cell stage embryos as reported previously [33].

### Western blot

Western blotting (WB) was performed as described previously [32, 34]. Embryos and cells were homogenized using Radio Immunoprecipitation Assay (RIPA) lysis buffer (Cat#P0013B, Beyotime Biotechnology), followed by adding an appropriate SDS-PAGE loading buffer, boiling, and loading for polyacrylamide gel electrophoresis. Next, the separated proteins were transferred to 0.45 μm nitrocellulose membrane (Millipore Corporation, Billerica, USA) for other WB procedures. The primary antibodies used in this study included Calnexin (AF5362, Affinity), Atf4 (10835-1-AP, Proteintech), Eif2α-pS51 (AF3087, Affinity), Hsf1 (ab242138, Abcam), E2F7 (A15211, ABclonal), E2F8 (A1135, ABclonal), Actin (AC026, ABclonal), and then with secondary antibody Goat anti-Rabbit lgG (H + L) in a 1:5000 dilution (Cat#BL033A, Biosharp, China).

### Bisulfite PCR validation

Control and CuNPs stressed, AgNPs stressed HUVECs and/or 50–60 embryos at 32 hpf were selected, separately, for DNA extraction and sulfurization using EZ DNA Methylation-Gold™ Kit (ZYMO RESEARCH, USA). JASPAR database (http://jaspar.gener.eg.net/) was used to predict the E2F7 binding site [5’-ATGAACCGC CAACT-3’] located at − 1886 bp to − 1873 bp, and E2F8 binding site [5’-TGAACCGCCAAC-3’] located at − 1885 bp to − 1874 bp in the *ccbe1* promoter; the E2F7 binding site [5’-AGTTATCGC CAAAG-3’] located at − 1956 bp to − 1943 bp, and E2F8 binding site [5’-GTTATCGCCAAA-3’] located at − 1955 bp to − 1944 bp in the *flt4* promoter from zebrafish; the E2F7 binding site [5’-ATTTGCCCCCACGT-3’] located at − 70 bp to − 57 bp, and E2F8 binding site [5’-TTTGCCCCC ACG − 3’] located at − 69 bp to − 58 bp in the *CCBE1* promoter; the E2F7 binding site [5’-TTTTGGCGAGGATG-3’] located at − 281 bp to − 268 bp, and E2F8 binding site [5’-TTTTTGGCGAGG − 3’] located at − 280 bp to − 271 bp in the *FLT4* promoter from human, which were also full of CpG sites, as we reported recently [14]. Target fragments were amplified using the specific primers (Supplementary Table 5) designed by Methyl Primer Express v1.0 (http://www.urogene.org/cgibin/methprimer/ methprimer. cgi). Bisulfite PCR products were separated and detected on 1% agarose gel, followed by purification using a Gel/PCR extraction kit (Omega Biotek, USA), sequencing, cloning into the pGEM-T Easy Vector (Promega, USA) and transformation into DH5α Chemically Competent Cell (Weidi, China). Finally, positive clones were identified by PCR and then sequenced for each fragment [7].

### Plasmid construction and injection

Genomic DNA was extracted from 50 to 60 whole embryos or HUVECs using the ammonium acetate method and quantified using a Nanodrop spectrophotometer (Thermo Fisher Scientific, USA) for gene promoter amplification. The primers in Supplementary Table 6 were used for amplification of the following regions (the − 2037/+ 403 of*ccbe1* promoter and − 2123/+ 150 of *flt4* promoter for zebrafish; − 131/+ 21 of *CCBE1* promoter and − 323/+ 238 of *FLT4* promoter for human, including the corresponding E2F7/8 binding sites), followed by cloning the amplified products separately into pCS2-GFP vector and pGL3 vector, as we reported recently [14]. All constructs were verified by sequencing. The constructed pCS2-GFP plasmids were diluted into 150 ng/µL and injected into one-cell stage embryos of wild type (WT) as reported previously [34]. The embryos injected with pCS2-GFP plasmid from the control group, CuNPs and AgNPs stressed groups at 52 hpf were anesthetized with a low dose of tricaine, and mounted on dishes with 1% low-melting agarose. Confocal images were captured by a Leica TCS SP8 confocal laser microscope (Wetzlar, Germany), and the fluorescence GFP in embryos was analyzed by software of Image J.

### Luciferase reporter assays

In this study, promoters of *CCBE1* and *FLT4* from zebrafish and/or human were used for luciferase assays to detect the biological effects of CuNPs and AgNPs on the transcriptional activities of zebrafish and human *CCBE1* and *FLT4* promoters, respectively. The plasmids were transiently transfected into HEK293T cells using Lipofectamine 2000 (Invitrogen, Carlsbad, CA, USA) following the manufacturer’s protocols. The luciferase reporter assays were performed as described previously [34]. All reporter plasmids were used in equimolar amounts in Opti-MEM (Invitrogen, USA), with the samples co-transfected with 20 ng pRL-TK used as control. After 4 h, the transfection medium was replaced by DMEM (10% FBS) in the control group, CuNPs stressed group, AgNPs stressed group. After 24 h incubation, cells were harvested to assay the luciferase activities by Dual-Luciferase Reporter Assay System (Promega, USA) following the manufacturer’s instruction. The data were reported as the mean ± SD of three independent experiments in triplicate.

### ChIP‑qPCR

ChIP-qPCR assays were performed as described previously [35]. Briefly, the input control, E2F7, E2F8, the IgG (negative control) samples of the control, CuNPs and AgNPs stressed groups were treated separately as we performed recently [32]. The tested genes (*CCBE1* and *FLT4*) in human cells and their primers used for ChIP-qPCR are listed in Supplementary Tables 7, and qPCR and data analysis were performed using recently reported methods [32, 35].

### Statistical analysis

The sample size for different experiments in each group was larger than 10 embryos (n > 10) with 3 biological replicates for each test. One-way analysis of variance [20] and post hoc Tukey’s test on GraphPad Prism 7.0 were used to analyze the data of qPCR, Western blot and ChIP-qPCR. All the experiments using HUVECs were repeated 3 times. The statistical significance between groups was determined at *P* < 0.05 (*), *P* < 0.01 (**) or *P* < 0.001 (***). Data are expressed as the mean ± standard deviation (SD) for normal distribution and median (range) for no-normal distribution.

## Results

### CuNPs and AgNPs stress impaired lymphangiogenesis *via* down-regulating *ccbe1* expression

**Fig. 1:**
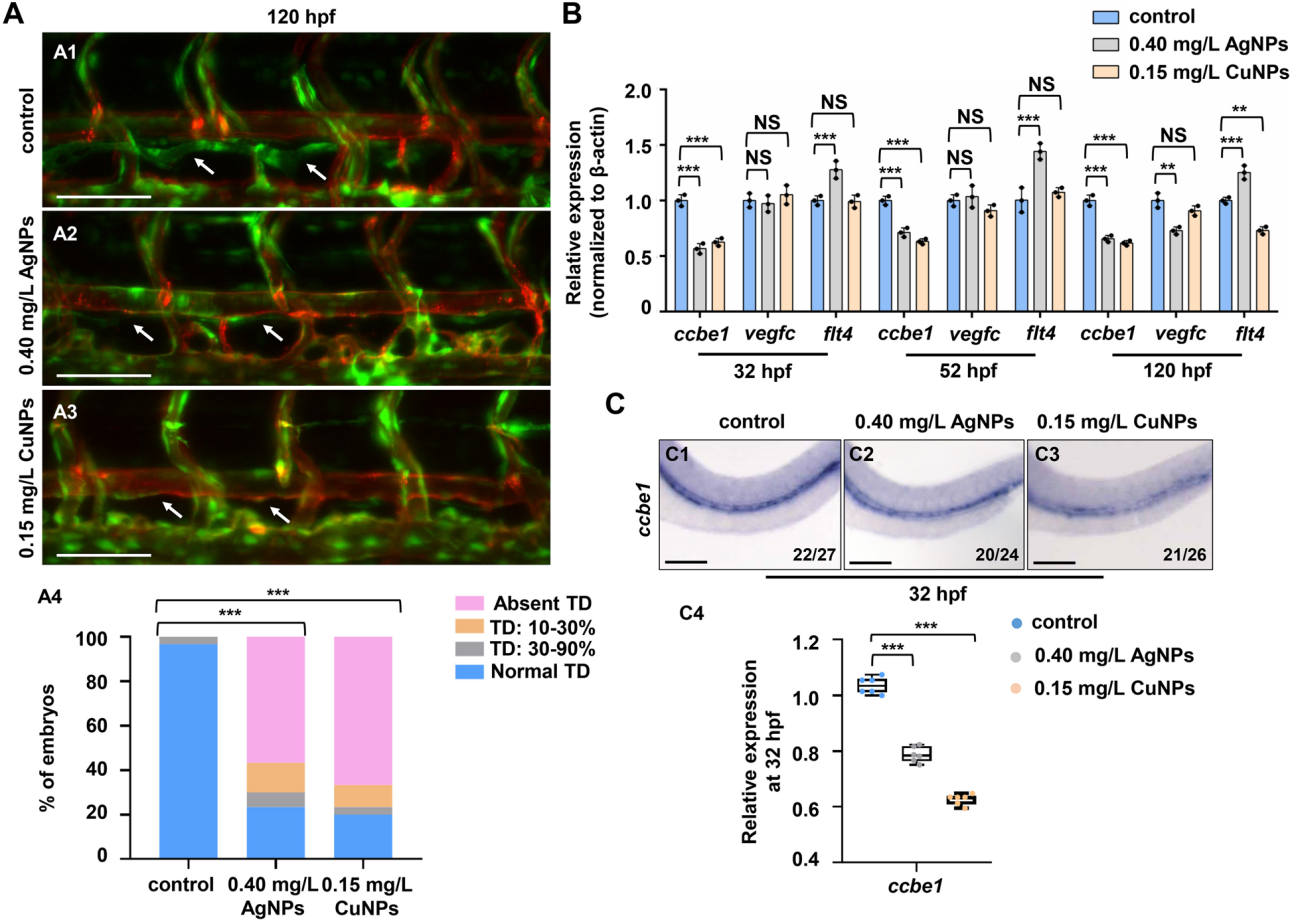
CuNPs and AgNPs stress impaired lymphangiogenesis *via* down-regulating ccbe1 expression. **A** Lateral images of lymphatics vessels of the control (A1), AgNPs-stressed (A2), and CuNPs-stressed (A3) Tg(*fli1a*:GFP; *flk1*:mCherry) zebrafish larvae at 120 hpf; blue arrows, *flk1* mCherry positive and *fli1a* GFP negative in lymphatics vessels (thoracic duct), and TD formation was quantitatively analyzed by measuring its presence from segments of somite 5 to somite 15 (A4).** B** RT-PCR expression analysis of lymphangiogenesis-related genes *ccbe1*, *vegfc*, and *flt4* in the control, AgNPs- and CuNPs-stressed embryos or larvae at 32 hpf, 52 hpf, and 120 hpf, respectively. **C** WISH analysis of gene *ccbe1* in the control, AgNPs- and CuNPs-stressed embryos at 32 hpf (C1 − C3), and quantitative analysis of WISH data for gene *ccbe1* in the three groups (C4). Data are mean ± SD. C1 − C3, lateral view, anterior to the left, and dorsal to the up. *P < 0.05, **P < 0.01, ***P < 0.001. NS, not significant. Scale bars, 100 μm (A1 − A3) and 500 μm (C1 − C3)

*Ccbe1* is essential in the development and formation of lymphatic vessels [25, 36], and we have reported that both Cu^2+^ and AgNPs stress can lead to the downregulation of *ccbe1* in 24 hpf and 96 hpf embryos of zebrafish [7], and Cu^2+^ stress induces lymphangiogenesis defects in zebrafish embryos *via* epigenetically down-regulating *ccbe1* expression [14]. In this study, the biological effects of CuNPs and AgNPs stress on zebrafish embryonic lymphangiogenesis were investigated. In CuNPs and AgNPs stressed Tg(*fli1a*:GFP; *flk1*:mCherry) embryos, the *fli1a*GFP^+^ *flk1*mCherry^−^ thoracic duct (TD), a crucial part of embryonic lymphatic structure was absent in approximately 67% CuNPs and 56% AgNPs stressed larvae at 120 hpf (Fig. 1A1 − A3). Significant reduction in the expression of *ccbe1* at 3 vital time points (32/52/120 hpf) of lymphangiogenesis and *flt4* at 120 hpf, but no significant change in the expression of *vegfc*, were observed in the CuNPs stressed embryos and larvae (Fig. 1B). Significant reduction in the expression of *ccbe1* at 32/52/120 hpf and *vegfc* at 120 hpf, was observed in the AgNPs stressed embryos and larvae. However, the expression of *flt4* was significantly increased at 32/52/120 hpf in the AgNPs stressed embryos and larvae (Fig. 1B). WISH assays further verified the reduction in the expression of *ccbe1* at 32 hpf in both CuNPs and AgNPs stressed embryos (Fig. 1C1 − C3).

#### AgNPs rather than CuNPs stress impaired the expression of transcription factors E2f7 and E2f8

**Fig. 2:**
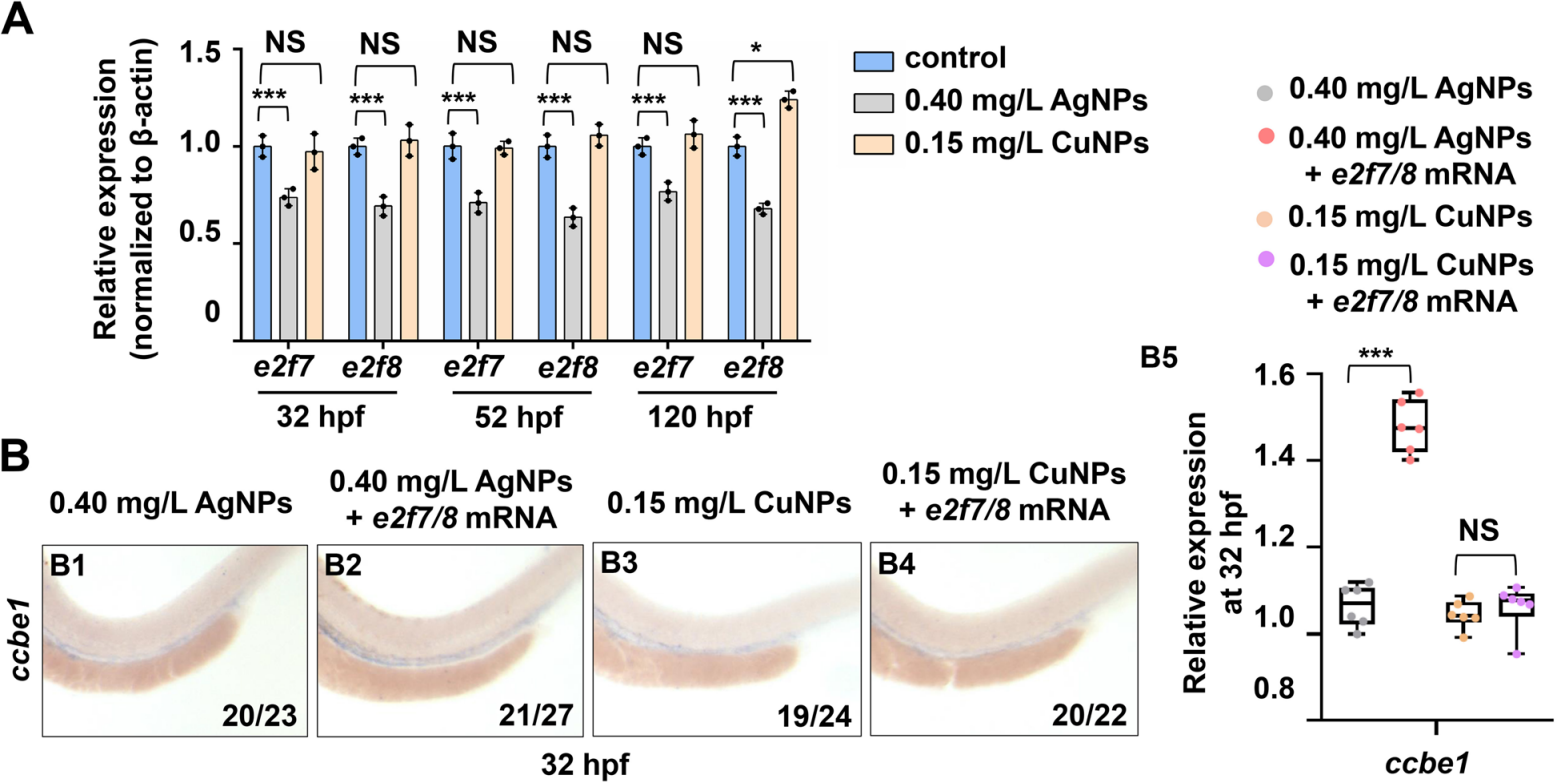
AgNPs rather than CuNPs stress impaired the expression of transcription factors E2f7 and E2f8. **A** RT-PCR expression analysis of genes *e2f7* and *e2f8* in the control, AgNPs- and CuNPs-stressed embryos or larvae at 32 hpf, 52 hpf, and 120 hpf, respectively. **B** WISH analysis of gene *ccbe1* in the AgNPs-stressed embryos, CuNPs-stressed embryos, AgNPs- and CuNPs-stressed embryos with ectopic expression of *e2f7* and *e2f8* at 32 hpf (B1 − B4), and quantitative analysis of WISH data for gene *ccbe1* in the four groups (B5). Data are mean ± SD. B1 − B4, lateral view, anterior to the left, and dorsal to the up. *P < 0.05, **P < 0.01, ***P < 0.001. NS, not significant. Scale bars, 500 μm (B1 − B4)

**Fig. 3:**
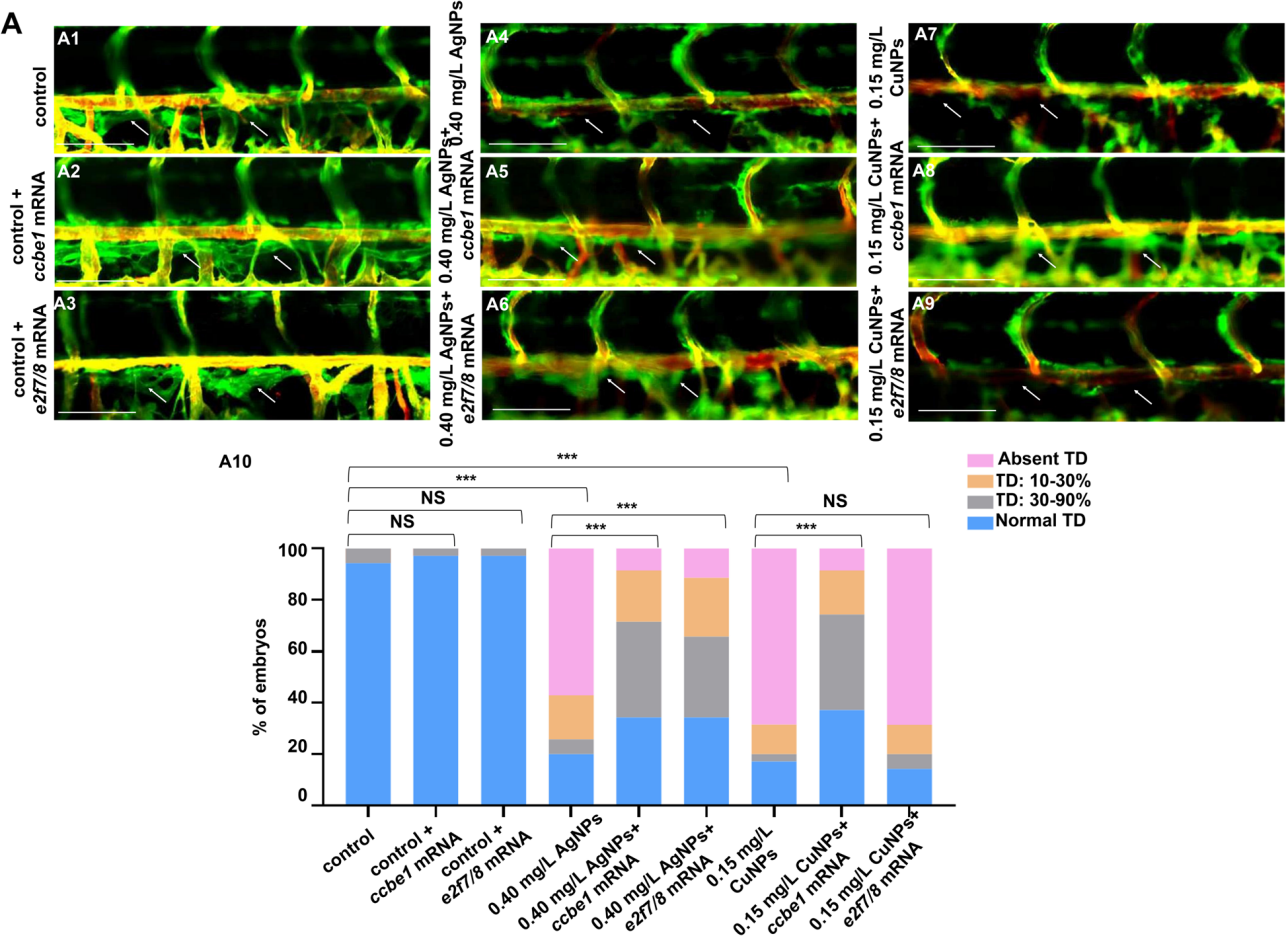
Salvage effect of *ccbe1/e2f7/e2f8* mRNA on lymphangiogenesis defects in the control, AgNPs- and CuNPs-stressed zebrafish larvae. A Lateral images of lymphatics vessels of the control (A1), AgNPs-stressed (A4), CuNPs-stressed (A7), control injected with *ccbe1* mRNA (A2), AgNPs group injected with *ccbe1* mRNA (A5), CuNPs group injected with *ccbe1* mRNA (A8), control injected with *e2f7/8* mRNA (A3), AgNPs group injected with *e2f7/8* mRNA (A6), CuNPs group injected with *e2f7/8* mRNA (A9) Tg(*fli1a*:GFP; *flk1*:mCherry) zebrafish larvae at 120 hpf; blue arrows, *flk1* mCherry positive and *fli1a* GFP negative in lymphatics vessels (thoracic duct), and TD formation was quantitatively analyzed by measuring its presence from segments of somite 5 to somite 15 (A10). Data are mean ± SD. *P < 0.05, **P < 0.01, ***P < 0.001. NS, not significant. Scale bars, 100 μm (A1 − A9)

Further, we detected the expression of transcription factors E2F7 and E2F8, which act up-stream of *CCBE1* and *FLT4* and bind their promoters directly, then to stimulate the expression of *CCBE1* but inhibit the expression of *FLT4* [14, 37]. In this study, *e2f7* and *e2f8* showed no significant change in transcriptional levels at the 3 vital time points (32/52/120 hpf) in the CuNPs stressed embryos and larvae, while showed significantly down-regulated in the AgNPs stressed group (Fig. 2A). Meanwhile, ectopic expression of *e2f7* and *e2f8* failed to rescue the down-regulated transcriptional expression of *ccbe1* at 32 hpf in CuNPs stressed embryos (Fig. 2B4) while effectively rescued the expression of *ccbe1* at 32 hpf in AgNPs stressed embryos (Fig. 2B2), suggesting AgNPs rather than CuNPs down-regulated *ccbe1* expression by impairing expressions of genes *e2f7* and *e2f8*, and CuNPs down-regulated *ccbe1* expression *via* other mechanisms. Overexpression of *e2f7/8* mRNA effectively rescued the lymphangiogenesis defects in AgNPs rather than CuNPs stressed larvae (Fig. 3). Meanwhile, overexpression of *ccbe1* mRNA effectively rescued the lymphangiogenesis defects in both AgNPs and CuNPs stressed larvae (Fig. 3).

### CuNPs stress induced hypermethylation of E2f7/8 binding sites on *ccbe1* promoter

**Fig. 4:**
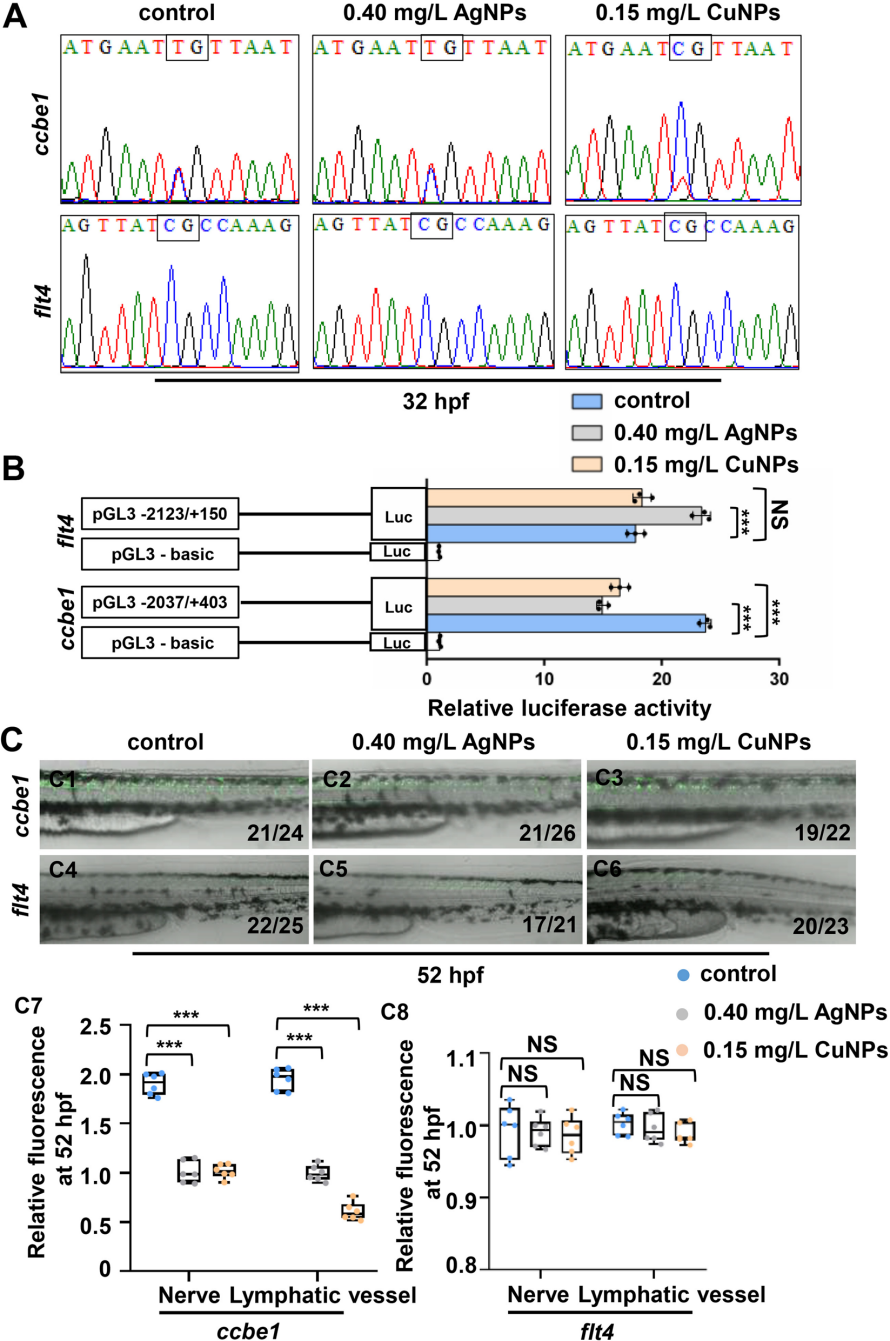
CuNPs stress induced hypermethylation of E2f7/8 binding sites on *ccbe1* promoter. **A** Methylations of CpG in E2F7/8 binding sites on *ccbe1* and *flt4* promoters of zebrafish embryos from the control, AgNPs- and CuNPs-stressed groups at 32 hpf. **B** Luciferase activities of zebrafish *ccbe1* or *flt4* promoter in the control, AgNPs- and CuNPs-stressed HEK293T cells, respectively. **C** The representative embryos injected with plasmids of *ccbe1* or *flt4* promoter-driven GFP from the control, AgNPs- and CuNPs-stressed groups at 52 hpf (C1 − C6), and quantitative analysis of fluorescence in nerves and lymphatic vessels in the representative embryos (C7 − C8). Data are mean ± SD. *P < 0.05, **P < 0.01, ***P < 0.001. NS, not significant. Scale bars, 250 μm (C1 − C6)

We have recently reported that Cu^2+^ stress hypermethylates the binding sites of transcriptional factors E2F7/8 on *CCBE1* promoter to down-regulate its expression, and the methylated loci is pivotal for the transcription of *CCBE1* [14]. Thus, in this study, in order to unveil how CuNPs stress induced the down-regulated expression of *ccbe1*, we further analyzed the relationship between *ccbe1* expression with its promoter DNA methylation of E2f7/8 binding sites in zebrafish embryos and larvae exposed to CuNPs or AgNPs, respectively. Compared to the control, CuNPs stressed embryos showed hypermethylation of CpG in E2f7/8 binding sites on *ccbe1* promoter, while no significant change in the methylation level on *flt4* promoter. The methylation level of CpG in E2f7/8 binding site on either *ccbe1* or *flt4* promoter of AgNPs stressed embryos did not change (Fig. 4A, S1B1). We recently have reported that the hypermethylation of *ccbe1* promoter and its down-regulated expression have been observed in both Cu^2+^ and AgNPs stressed embryos [7], and the altered methylation sites on *ccbe1* promoter induced by Cu^2+^ stress was also observed in CuNPs stressed rather than in AgNPs stressed embryos in this study. Meanwhile, the altered methylation sites on *ccbe1* promoter induced by AgNPs stress [7] were not detected in this study.

**Fig. 5:**
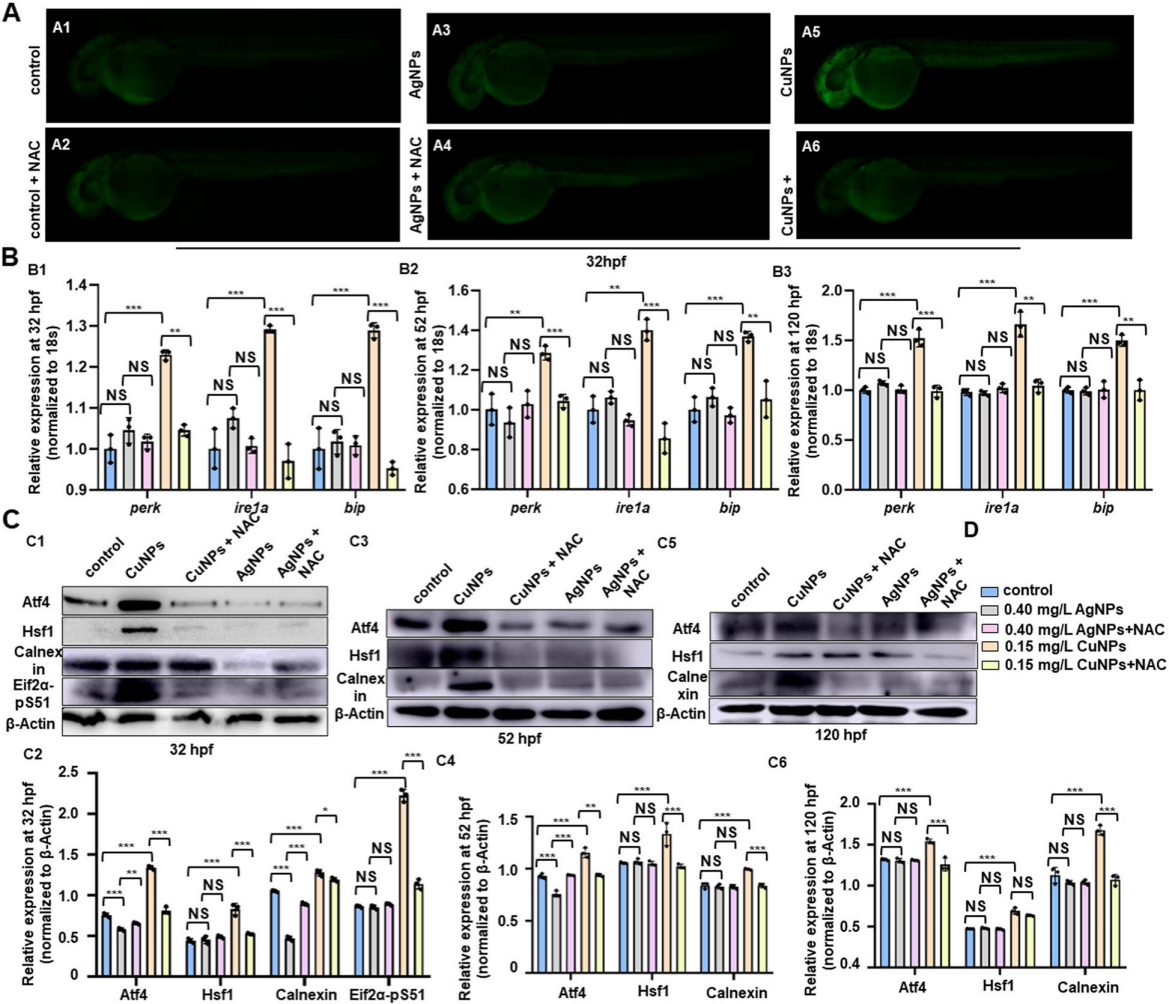
CuNPs rather than AgNPs stress induced ROS stresses in zebrafish embryos. **A** Reactive oxygen species levels in the control (A1), AgNPs- (A3) and CuNPs-stressed embryos (A5) and in the corresponding ROS scavenger NAC co-treated individuals (A2, A4, A6) by DCFH-DA staining. **B** RT-PCR expression analysis of genes *perk*, *ire1a*, *bip* in the control, AgNPs- and CuNPs-stressed embryos or larvae and in the corresponding NAC co-treated individuals (B1 − B3). **C** Protein levels of Atf4, Hsf1, Calnexin and Eif2a-pS51 in the control, AgNPs- and CuNPs-stressed embryos or larvae and in the corresponding NAC co-treated individuals (C1, C3, C5), and quantitative analysis of protein level in embryos or larvae from the five groups respectively (C2, C4, C6). **D** B-C legend. Data are mean ± SD. A1 − A6, lateral view, anterior to the left, and dorsal to the up. *P < 0.05, **P < 0.01, ***P < 0.001. NS, not significant

Next, we detected the transcriptional activities of *ccbe1* and *flt4* promoters after CuNPs or AgNPs stress by double luciferase reporter assays. CuNPs stress significantly reduced the luciferase activities of *ccbe1* promoter (pGL3 − 2037/+ 403), but did not significantly change the luciferase activities of *flt4* promoter (pGL3 − 2123/+ 150). Meanwhile, AgNPs stress significantly reduced the luciferase activities of *ccbe1* (pGL3 − 2037/+ 403) while increased the luciferase activities of *flt4* promoter (pGL3 − 2123/+ 150) (Fig. 4B). The results here indicated that the − 2037/+ 403 region of *ccbe1* promoter was negatively responsive to both CuNPs and AgNPs stress, while the − 2123/+ 150 region of *flt4* promoter was insensitive to CuNPs stress but positively sensitive to AgNPs stress.

Similarly, compared with the control group, the GFP fluorescence in nerves and lymphatic vessels driven by *ccbe1* promoter at 52 hpf was significantly reduced in the injected embryos stressed with either CuNPs or AgNPs, respectively (Fig. 4C2, C3, C7), while the GFP fluorescence in nerves and lymphatic vessels driven by *flt4* promoter was not significantly changed at 52 in the CuNPs stressed embryos and larvae (Fig. 4C5, C6, C8), which was consistent with the results of luciferase reporter assays. Additionally, compared with the control group, the GFP fluorescence in nerves driven by *ccbe1* promoter at 120 hpf was significantly reduced in the injected larvae stressed with either CuNPs or AgNPs, respectively (Figs. S1A2, A3, A7), while the GFP fluorescence in nerves driven by *flt4* promoter was not significantly changed at 120 hpf in the CuNPs stressed larvae (Figs. S1A6, A7), but obviously up regulated at 120 hpf in the AgNPs stressed larvae (Figs. S1A5, A7).

#### CuNPs rather than AgNPs stress induced ROS stresses in zebrafish embryos

Previous studies have shown that CuNPs could cause intestinal developmental defects *via* inducing ER and ROS stresses [31]. Sustained and excessive cellular ROS levels resulted in genetic as well as epigenetic alterations [38]. More specifically, ROS production is associated with alterations in DNA methylation patterns [39, 40]. Thus, we further investigated the ROS level in the embryos or larvae from the control group, CuNPs and AgNPs stressed group and individuals from the corresponding NAC treated group by DCFH-DA staining in this study. The results showed that the ROS levels of zebrafish embryos were significantly up-regulated at 32 hpf in the CuNPs stressed group (Fig. 5A5) while with no significant change in the AgNPs stressed group (Fig. 5A3). Additionally, NAC could restore the increased ROS level in CuNPs stressed embryos to nearly normal level (Fig. 5A6). Next, the present study detected the expression of stress markers *perk*, *ire1a*, *bip* (which down-stream of ROS and could be induced by increased ROS [31, 41–43]) at the 3 vital time points (32/52/120 hpf) in the embryos and larvae from the control, CuNPs stressed, and AgNPs stressed and the corresponding NAC treated groups, and the results showed that the expressions of genes *perk*, *ire1a*, *bip* were significantly increased in CuNPs stressed embryos and larvae (Fig. 5B1 − B3), and NAC could restore the increased expression of the genes in CuNPs stressed embryos and larvae to nearly normal level, but there were no significant changes in the expression of the genes in AgNPs stressed embryos and larvae. Additionally, the increased protein levels of stress markers Calnexin, Atf4, Eif2a-pS51 and Hsf1 were observed in CuNPs stressed embryos and larvae (Fig. 5C1 − C6), and NAC could restore the increased expression of the proteins in CuNPs stressed embryos and larvae to nearly normal level. Differently, the expression of Calnexin and Atf4 were significantly down-regulated at 32 hpf (Fig. 5C1, C2) and Atf4 down-regulated at 52 hpf in AgNPs stressed embryos (Fig. 5C3, C4).

### CuNPs stress induced ROS mediated hypermethylation of the E2F7/8 binding site on *CCBE1* promoter

**Fig. 6:**
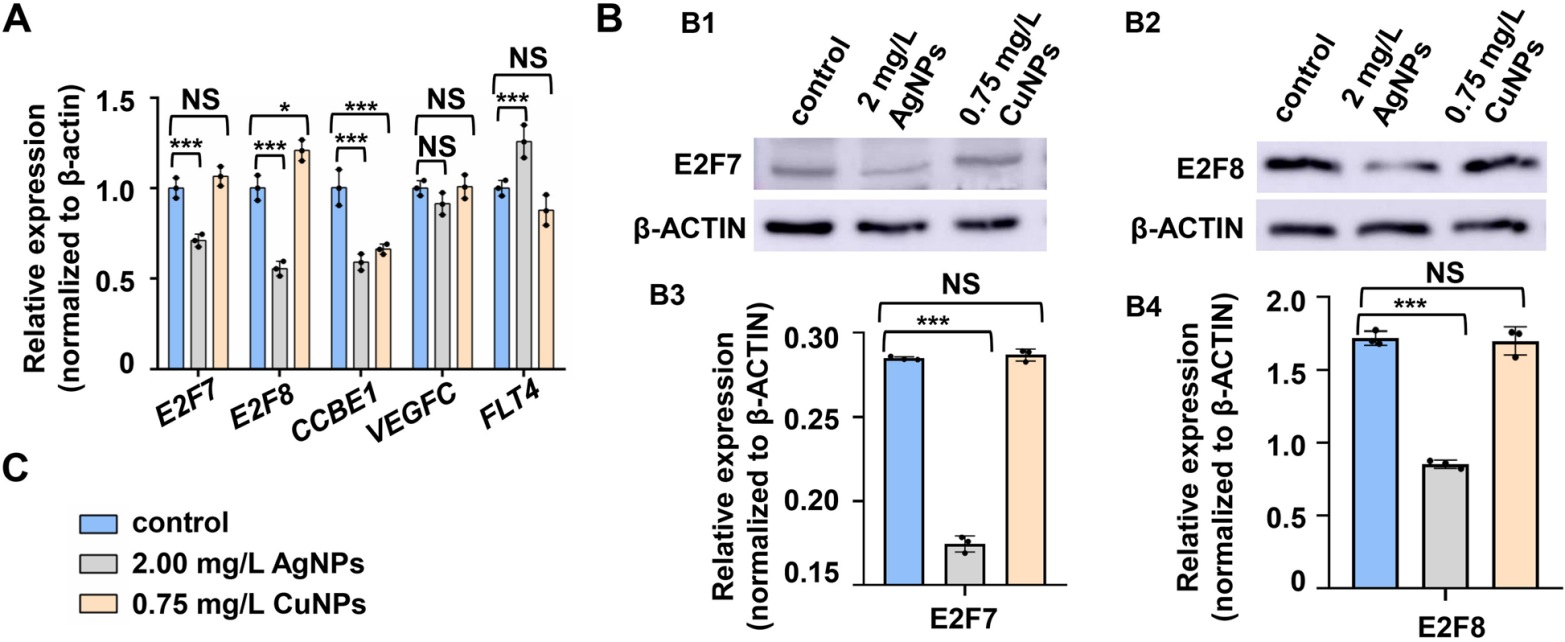
AgNPs rather than CuNPs stress impaired the expression of transcription factors E2F7 and E2F8 in HUVECs. **A** RT-PCR expression analysis of genes *E2F7*, *E2F8*, *CCBE1*, *VEGFC*, and *FLT4* in CuNPs- and AgNPs-stressed HUVECs relative to their expression in the control cells. **B** Protein levels of E2F7 and E2F8 in the control, AgNPs- and CuNPs-stressed HUVECs (B1, B2), and quantitative analysis of protein level in cells from the three groups (B3, B4). **C** A-B legend. Data are mean ± SD. *P < 0.05, **P < 0.01, ***P < 0.001. NS, not significant

We next investigated whether expression of *CCBE1* was also inhibited by CuNPs or AgNPs stress in human cells, and investigated whether the CuNPs produced ROS might induce the hypermethylation of E2F7/8 binding sites on *CCBE1* promoter. In this study, the transcriptional expression of *CCBE1* was significantly reduced in CuNPs and AgNPs stressed HUVECs relative to that in the control cells. The transcriptional expressions of *E2F7* and *E2F8* were significantly decreased and the expression of *FLT4* was significantly up-regulated in AgNPs stressed cells, but *E2F7*, *E2F8*, *VEGFC*, and *FLT4* showed no difference in the transcriptional levels in CuNPs stressed cells (Fig. 6A). Compared with their protein levels in the control cells, the protein level of E2F7 and E2F8 showed no change in CuNPs stressed HUVECs while were significantly decreased in AgNPs stressed cells (Fig. 6B1 − B4).

**Fig. 7:**
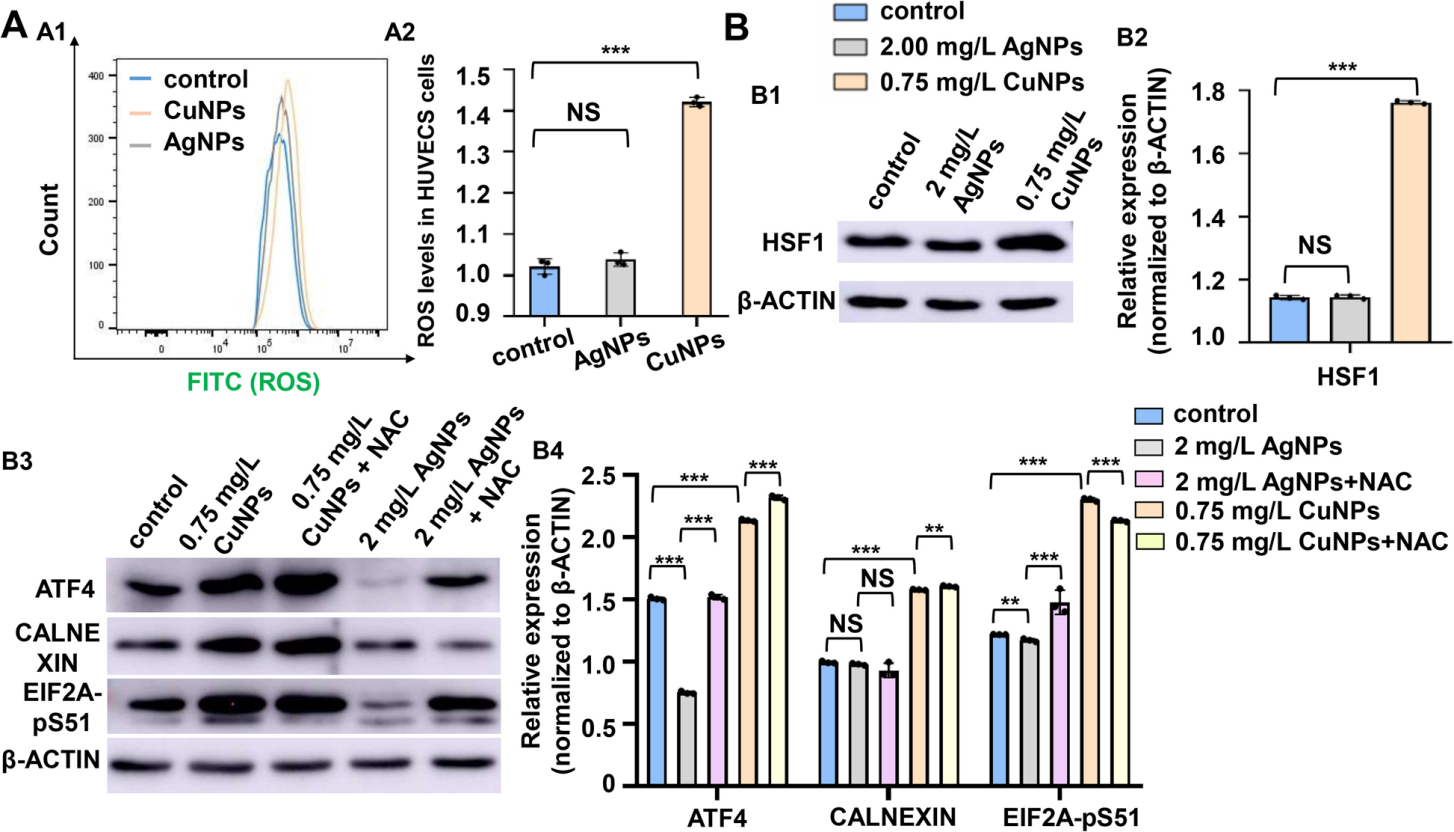
CuNPs rather than AgNPs stress induced ROS stresses in HUVECs. **A** Reactive oxygen species levels in the control, AgNPs- and CuNPs-stressed HUVECs (A1) by DCFH-DA staining, and quantitative analysis of ROS level in cells from the three groups (A2). **B** Protein levels of HSF1 in the control, AgNPs- and CuNPs-stressed HUVECs (B1); protein levels of ATF4, CALNEXIN and EIF2A-pS51 in the control, AgNPs- and CuNPs-stressed HUVECs and the corresponding NAC treated HUVECs (B3), and quantitative analysis of protein level in cells of the five groups respectively (B2, B4). Data are mean ± SD. *P < 0.05, **P < 0.01, ***P < 0.001. NS, not significant

ROS levels increased in the CuNPs stressed HUVECs while with no significant difference in the AgNPs stressed cells (Fig. 7A1, A2). The increase in the protein levels of stress indicators CALNEXIN, ATF4, EIF2A-pS51, and HSF1 were observed in CuNPs stressed HUVECs (Fig. 7B1 − B4), and ROS scavenger NAC effectively restored their increased expressions to nearly normal level. Differently, the expressions of CALNEXIN, ATF4 and EIF2A-pS51 were significantly down-regulated in AgNPs stressed HUVECs (Fig. 7B3, B4).

**Fig. 8:**
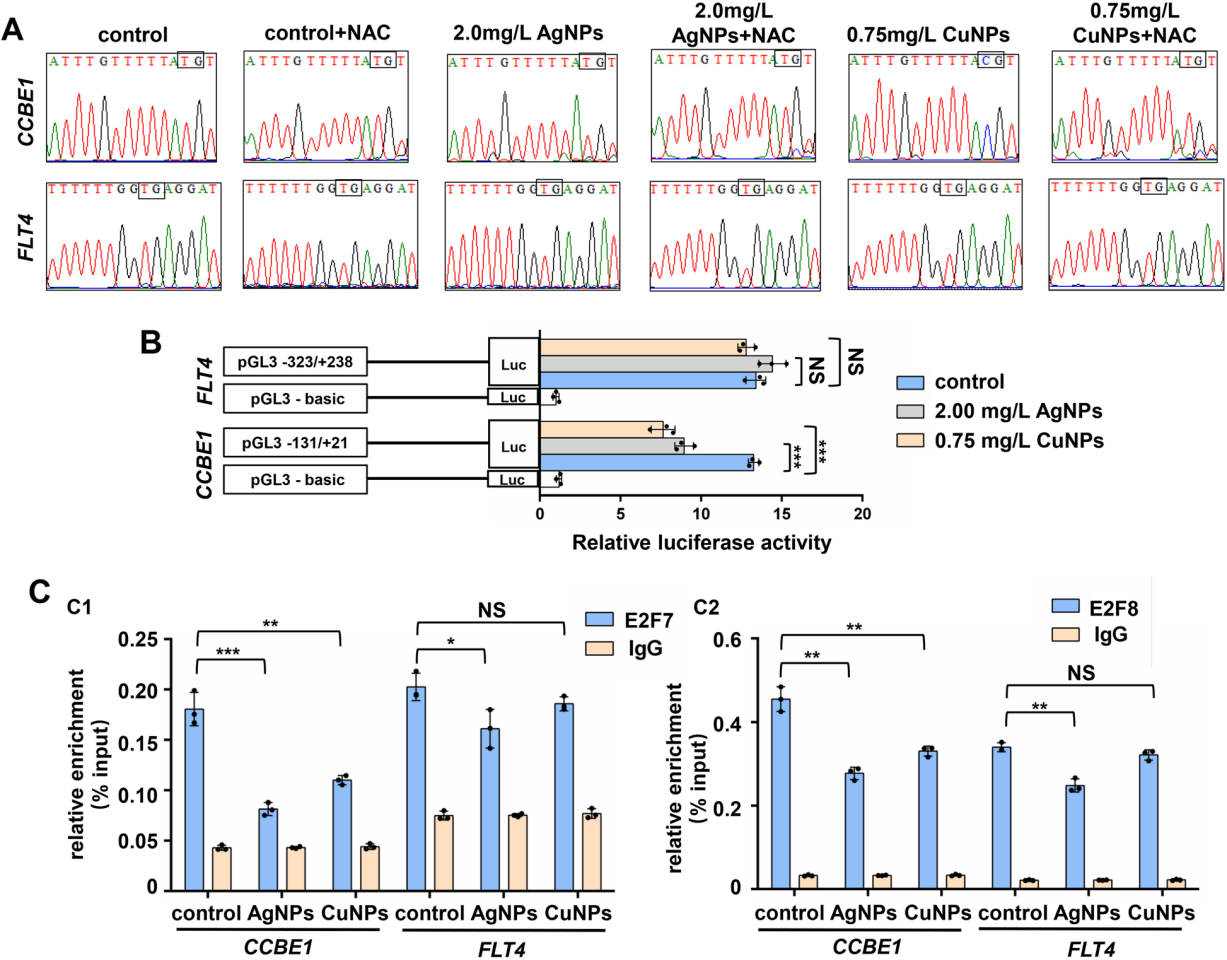
CuNPs stress induced hypermethylation of E2F7/8 binding sites on *CCBE1* promoter in HUVECs. **A** Methylations of E2F7/8 binding sites on *CCBE1* and *FLT4* promoters in the control, AgNPs- and CuNPs-stressed HUVECs and the corresponding NAC treated HUVECs. **B** Luciferase activities of human *CCBE1* and *FLT4* promoters in the control, AgNPs- and CuNPs-stressed HEK293T cells, respectively. **C** Binding enrichment of proteins E2F7 (C1) and E2F8 (C2) for human *CCBE1* and *FLT4* promoters as revealed by chromatin immunoprecipitation assays (ChIP) in the control, AgNPs- and CuNPs-stressed HUVECs. Data are mean ± SD. *P < 0.05, **P < 0.01, ***P < 0.001. NS, not significant

Compared to the control, CuNPs stressed HUVECs exhibited CpG hypermethylation in the E2F7/8 binding sites on *CCBE1* promoter, while no significant change in the CpG methylations on *FLT4* promoter (Fig. 8A, S1B2). In addition, NAC could restore the CpG hypermethylation level of *CCBE1* promoter to normal, strongly indicating that CuNPs stress induced ROS mediated the hypermethylation of the E2F7/8 binding site on *CCBE1* promoter. However, CpG methylation levels on *CCBE1* and *FLT4* promoters did not change significantly in AgNPs stressed group. Moreover, CuNPs and AgNPs stress significantly reduced the luciferase activities of *CCBE1* promoter (pGL3 − 131/ + 21), but did not significantly change the luciferase activities of *FLT4* promoter (pGL3 − 323/ + 238), which was consistent with the results in zebrafish (Fig. 8B).

Further binding enrichment analysis indicated that, compared with the control, CuNPs stressed HUVECs showed significant reduction in the binding enrichment of protein E2F7 and E2F8 on *CCBE1* promoter, but no significant change on *FLT4* promoter. While AgNPs stressed HUVECs showed significant reduction in the binding enrichment of protein E2F7 and E2F8 on *CCBE1* promoter and *FLT4* promoter (Fig. 8C1, C2), indicating that *CCBE1* is inhibited by both CuNPs and AgNPs stress.

## Discussion

The vascular system consists of blood vessels and lymphatics. Lymphatic vessels are mainly divided into lymphatic vessels, lymphatic ducts and capillary lymphatic vessels, among which the thoracic duct is the largest lymphatic vessel discharging lymph into the blood circulation [44]. In this study, developmental defects of the thoracic duct are found in both CuNPs and AgNPs stressed zebrafish embryos, which is similar to the phenotype of lymphangiogenesis defects in *ccbe1* mutants and Cu^2+^ stressed embryos in zebrafish [36, 44]. This might be the first to demonstrate that nanomaterials induce lymphangiogenesis defects in vertebrates. We have recently reported that Cu^2+^ stress induces lymphangiogenesis defects in zebrafish larvae [14], and reported that CuNPs exert their biological effects *via* their released ions [45, 46]. Thus, we speculate that the released Cu^2+^ ions might mediate CuNPs stress induced lymphangiogenesis defects during zebrafish embryogenesis in this study. However, some assays are needed to do in the further days to test whether AgNPs damage lymphangiogenesis *via* their released ions.

The CCBE1/VEGFC/FLT4 signaling pathway is essential for lymphangiogenesis [28]. VEGFC has been reported to require two proteolytic processing steps to bind to its receptor and initiate the sprouting process [47], and the second cleavage is mediated by CCBE1 to release the fully active form of VEGFC, which then binds to and activates the FLT4 receptor in lymphoid precursor cells to induce lymphangiogenic cell behavior. In this study, the expression of *CCBE1* is decreased (32/52/120 hpf) and the expression of *VEGFC* is not changed in both CuNPs stressed embryos and mammalian cells, and in AgNPs stressed embryos, and overexpression of *ccbe1* mRNA effectively rescued the lymphangiogenesis defects in both CuNPs and AgNPs stressed embryos, suggesting the down-regulated expression of *CCBE1* might be the potential contributor underlying the lymphangiogenesis defects occurred in both CuNPs and AgNPs stressed embryos, and the roles of CuNPs and AgNPs stress in down-regulating *CCBE1* expression are conserved from fish to mammalian cells.

E2F7 and E2F8 have been reported to bind directly on the *CCBE1* or *FLT4* promoter and positively stimulate the expression of *CCBE1* but inhibit the expression of *FLT4* [37]. Interestingly, consistent with the results in mammalian cells, during lymphangiogenesis, the expression of E2F7 does not change in the CuNPs stressed embryos, but the expression of E2F8 (120 hpf) is slightly upregulated, which may be the reason for the decreased expression of *FLT4* (120 hpf) in the CuNPs stressed embryos. Moreover, overexpression of *E2F7* and *E2F8* fails to recuse the expression of *CCBE1* in CuNPs stressed embryos. All these observations indicate that CuNPs stress down-regulates the expression of *CCBE1 via* other mechanisms rather than down-regulating the expression of the *E2F7/8*. CuNPs has been reported to cause intestinal developmental defects *via* inducing ER and ROS stresses [45], consistently, the ROS stress level and the protein levels of stress indicators are significantly up-regulated in both zebrafish embryos and HUVECs after CuNPs stress but not after AgNPs stress, and ROS scavenger NAC could effectively restore their increased levels, suggesting CuNPs stress rather than AgNPs stress induces ROS in the stressed embryos and HUVECs.

Sustained and excessive cellular ROS levels lead to genetic as well as epigenetic alterations [38]. More specifically, ROS production is associated with alterations in DNA methylation patterns [39, 40]. Consistently, in this study, CuNPs stressed embryos and mammalian HUVECs exhibit hypermethylation of E2F7/8 binding sites on *CCBE1* promoter, in contrast to no change on *FLT4* promoter, similar to previous observations [14]. Differentially, the E2F7/8 binding sites on *CCBE1* and *FLT4* promoters exhibit no methylated change in AgNPs stressed embryos and mammalian cells. CuNPs stress induces the significant down-regulation in the transcriptional activities of *CCBE1* promoter in both zebrafish and mammalian cells, while the mRNA and protein levels of E2F7 and E2F8 remain unchanged in CuNPs stressed embryos and HUVECs, but the binding enrichments of E2F7 and E2F8 are significantly reduced on the *CCBE1* promoter rather than on the *FLT4* promoter, suggesting CuNPs stress might impair *CCBE1* transcriptional activity *via* its induced hypermethylated E2F7/8 binding loci to affect E2F7/8 binding on *CCBE1* promoter, then to down-regulate *CCBE1* expression, rather than *via* changing E2F7 and E2F8 expression directly. The observations here are consistent with the recent report that Cu^2+^ stress induces lymphangiogenesis defects in zebrafish embryos *via* epigenetically regulating E2F7/8 transcriptional activities on *ccbe1* expression [14]. In addition, NAC could restore the CpG hypermethylation level of *CCBE1* promoter to normal, strongly indicating that CuNPs stress induced ROS mediates hypermethylation of the E2F7/8 binding site on *CCBE1* promoter. However, the molecular mechanism of ROS mediated site-specific methylation on *CCBE1* promoter still needs further study.

Differentially, in the AgNPs stressed embryos and HUVECs, significant reduction in the expression of *CCBE1* but significantly up-regulation in the expression of *FLT4* are unveiled. In addition, the expressions of E2F7 and E2F8 is significantly down-regulated in the AgNPs stressed embryos and HUVECs, and overexpression of *e2f7* and *e2f8* could restore the expression of *ccbe1* in the AgNPs stressed embryos and rescue the lymphangiogenesis defects in AgNPs stressed embryos, suggesting AgNPs stress down-regulates *CCBE1* expression *via* down-regulating *E2F7* and *E2F8* expression directly, then lead to lymphangiogenesis defects in AgNPs stressed embryos. The E2F7/8 binding sites on *CCBE1* and *FLT4* promoters are not methylated in AgNPs stressed embryos and mammalian cells, while the binding enrichments of E2F7 and E2F8 are significantly reduced on the *CCBE1* promoter and on the *FLT4* promoter, which may be contributed to the down-regulated expression of *CCBE1* and the up-regulated expression of *FLT4*, which is consistent with the point that E2F7 and E2F8 positively stimulate the expression of *CCBE1* but inhibit the expression of *FLT4* [37]. The observations here indicate that AgNPs down-regulates *CCBE1* expression by inhibiting the expression of *E2F7* and *E2F8* directly. However, it has been reported that AgNPs stress could lead to hypermethylation of *ccbe1* promoter in previous study [7], but the candidate hypermethylated site on *ccbe1* promoter tested in this study do not show hypermethylation in AgNPs stressed cells, suggesting the methylation may occur at other sites, which needs further detection.

## Conclusions

**Fig. 9:**
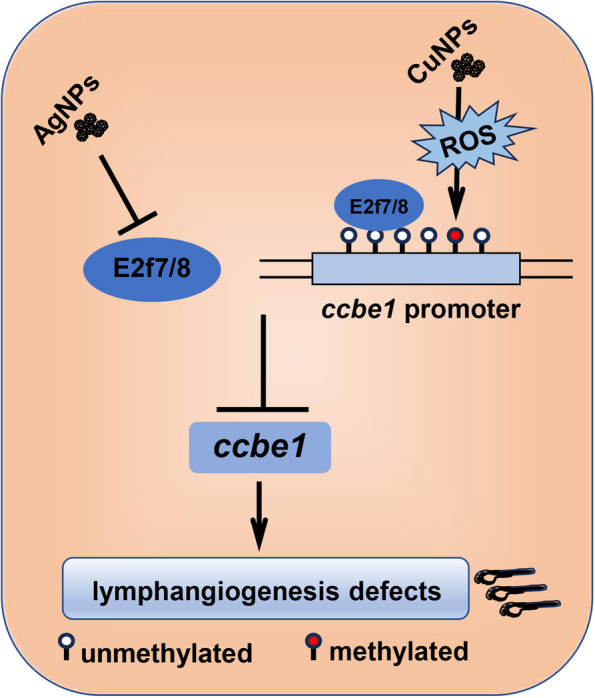
Working model of AgNPs and CuNPs stress in inducing lymphangiogenesis defects. CuNPs overload induced lymphangiogenesis defects in zebrafish *via* hypermethylation of E2f7/8 binding sites on ccbe1 promoter through their induced ROS, while AgNPs overload via down-regulating E2f7/8 expressions

Overall, this study confirmed that CuNPs stress down-regulates *CCBE1* expression by producing ROS stress to induce the hypermethylation of E2F7/8 binding sites on *CCBE1* promoter, thereby leading to the reduced binding enrichments of E2F7/8 on *CCBE1* promoter and its reduced expression, then resulting in lymphangiogenesis defects. However, AgNPs down-regulate the expression of *CCBE1* by inhibiting the expression of E2F7/8 directly, then leading to the reduced expression of their downstream gene *CCBE1*, thus inducing lymphangiogenesis defects. Overexpression of *ccbe1* mRNA effectively rescued the lymphangiogenesis defects in both AgNPs and CuNPs stressed larvae, while overexpression of *e2f7/8* mRNA effectively rescued the lymphangiogenesis defects in AgNPs rather than CuNPs stressed larvae. Meanwhile, we also cannot rule out the possibility that other untested genes or signals might mediate CuNPs and AgNPs stress induced lymphangiogenesis defects. The working model is illustrated in Fig. 9 for an intuitive understanding of how CuNPs and AgNPs stress induces lymphangiogenesis defects during zebrafish embryogenesis. However, based on our current observations, we still have not detected the methylated site on *ccbe1* promoter induced by AgNPs stress, nor have we been able to thoroughly establish the molecular mechanism model of ROS induced site-specific methylation on gene promoters. At the same time, we also cannot rule out the possibility that other untested genes or signals might mediate CuNPs and AgNPs stress induced lymphangiogenesis defects.

### Supplementary Information

**Additional file 1: Supplementary Fig 1.** The sequencing results of positive clones for each fragment including the E2F7/8 binding sites on *CCBE1* promoter in the embryos or HUVECs. A The representative embryos injected with plasmids of *ccbe1* or *flt4* promoter-driven GFP from the control, AgNPs- and CuNPs-stressed groups at 120 hpf (A1−A6), and quantitative analysis of fluorescence in nerves in the representative embryos (A7). B The sequencing results of positive clones for each fragment in the control, AgNPs- and CuNPs-stressed embryos (B1) or HUVECs (B2). Data are mean ± SD. *P < 0.05, **P < 0.01, ***P < 0.001. NS, not significant. Scale bars, 250 μm (A1−A6). **Table S1.** List of genes tested in this study. **Table S2**. Sequences of primers used for qRT-PCR. **Table S3.** Sequences of primers used for amplifying probes for WISH. **Table S4.** Sequences of primers for mRNA synthesis. **Table S5.** Sequences of primers used for promoter methylation level assays. **Table S6.** Sequences of primers used for promoter activity assays. **Table S7.** Sequences of primers used for ChIP-qPCR.
