# Supplementary material for: Copper nanoparticles and silver nanoparticles impair lymphangiogenesis in zebrafish
Source: Cell Commun Signal. 2024 Jan 25;22:67. doi: 10.1186/s12964-023-01403-x (PMC10809531; doi:10.1186/s12964-023-01403-x)
Supplement: Supplementary file 2 — Additional file 1: Supplementary Fig 1. The sequencing results of positive clones for each fragment including the E2F7/8 binding sites on CCBE1 promoter in the embryos or HUVECs. A The representative embryos injected with plasmids of ccbe1 or flt4 promoter-driven GFP from the control, AgNPs- and CuNPs-stressed groups at 120 hpf (A1−A6), and quantitative analysis of fluorescence in nerves in the representative embryos (A7). B The sequencing results of positive clones for each fragment in the control, AgNPs- and CuNPs-stressed embryos (B1) or HUVECs (B2). Data are mean ± SD. *P < 0.05, **P < 0.01, ***P < 0.001. NS, not significant. Scale bars, 250 μm (A1−A6). Table S1. List of genes tested in this study. Table S2. Sequences of primers used for qRT-PCR. Table S3. Sequences of primers used for amplifying probes for WISH. Table S4. Sequences of primers for mRNA synthesis. Table S5. Sequences of primers used for promoter methylation level assays. Table S6. Sequences of primers used for promoter activity assays. Table S7. Sequences of primers used for ChIP-qPCR. [file 12964_2023_1403_MOESM1_ESM.docx]

**
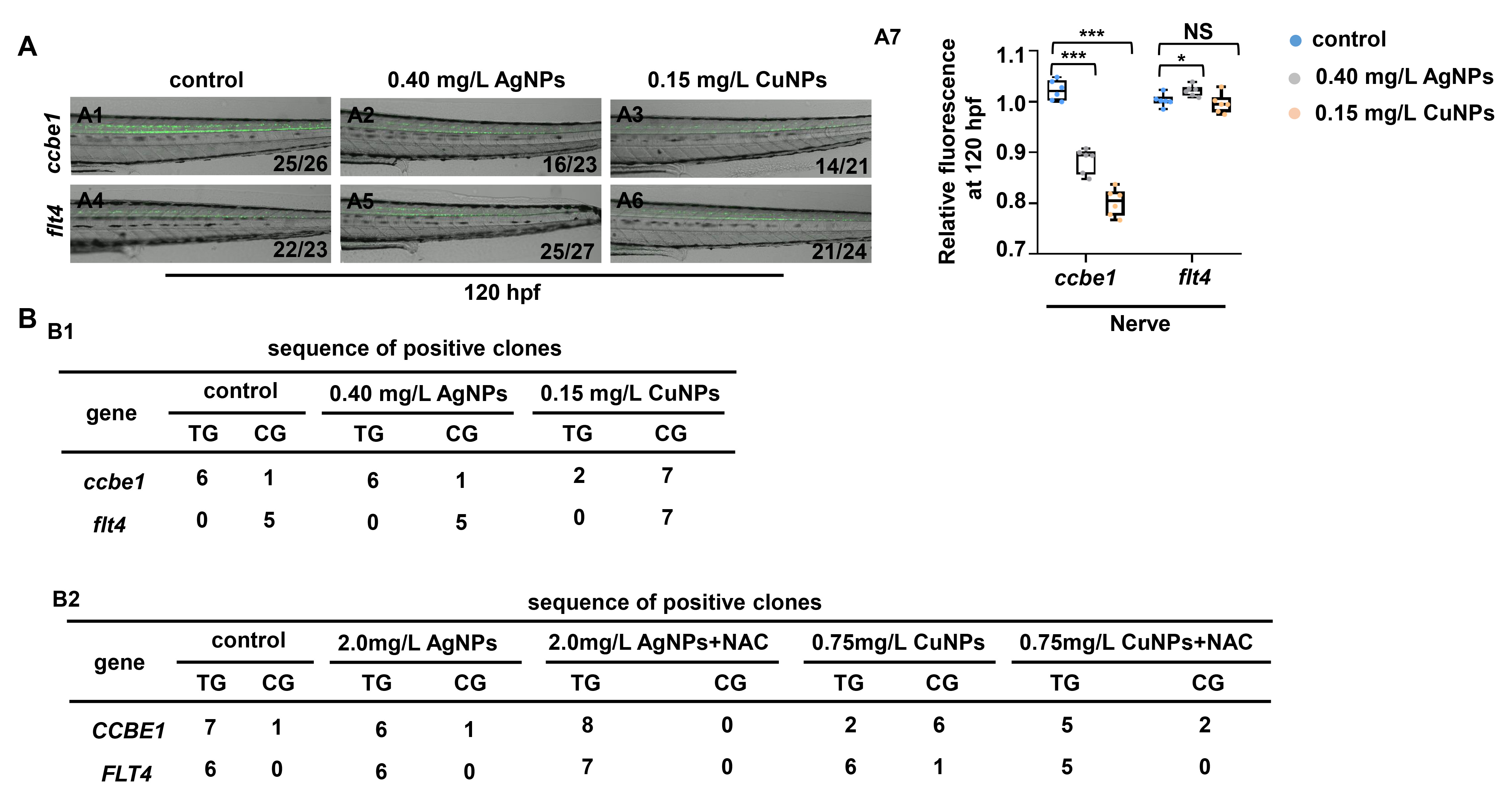
**

**Supplementary Fig 1 The sequencing results of positive clones for each fragment including the E2F7/8 binding sites on *CCBE1* promoter in the embryos or HUVECs.** A The representative embryos injected with plasmids of *ccbe1* or *flt4* promoter-driven GFP from the control, AgNPs- and CuNPs-stressed groups at 120 hpf (A1−A6), and quantitative analysis of fluorescence in nerves in the representative embryos (A7). B The sequencing results of positive clones for each fragment in the control, AgNPs- and CuNPs-stressed embryos (B1) or HUVECs (B2). Data are mean ± SD. **P* < 0.05, ***P* < 0.01, ****P* < 0.001. NS, not significant. Scale bars, 250 μm (A1−A6).

| **Table S1. List of genes tested in this study** | |  |
| --- | --- | --- |
| Gene symbol | Full names |  |
|  |  |  |
| *perk* | eukaryotic translation initiation factor 2 alpha kinase 3 |  |
| *ire1a* | tumor protein p53 |  |
| *bip* | heat shock protein 5 |  |
| *flk1* | kinase insert domain receptor like |  |
| *fli1* | Fli-1 proto-oncogene, ETS transcription factor |  |
| *ccbe1* | collagen and calcium-bind-ing EGF domains 1 |  |
| *flt4* | fms-related receptor tyrosine kinase 4 |  |
| *vegfc* | vascular endothelial growth factor C |  |
| *e2f7* | E2F transcription factor 7 |  |
| *e2f8* | E2F transcription factor 8 |  |
| Atf4 | Activating Transcription Factor 4 |  |
| Hsf1 | Heat shock factor 1 |  |
| Eif2α-pS51 | phosphorylation of Eif2a |  |

| **Table S2. Sequences of primers used for qRT-PCR** | | |  |
| --- | --- | --- | --- |
| gene | Primer sequence(5'to3') | GenBank ID |  |
|  |  |  |  |
| *bip* (zebrafish) | F: 5' ATCAGATCTGGCCAAAATGC 3' | NM_213058.1 |  |
|  | R: 5' CCACGTATGACGGAGTGATG 3' |  |  |
| *ire1a* (zebrafish) | F: 5' ATGGCGTGGGGAGTGTGC 3' | NM_001328588.1 |  |
|  | R: 5' GTATTCTGTGCGGCCAAGGTAAA 3' |  |  |
| *perk* (zebrafish) | F: 5' CCGCGGGGCAACAGAGT 3' | XM_005156585.4 |  |
|  | R: 5' GGTGGCAGCGATACAGAAGAAGAT 3' |  |  |
| *flt4* (zebrafish) | F: 5' TGCACCAGTATGCCACATTT 3' | AY833404.1 |  |
|  | R: 5' TGCTTCCATTGCTTTGACTG 3' |  |  |
| *vegfc* (zebrafish) | F: 5' AGCGGGTAGTGTGGATGAAC 3' | NM_205734.1 |  |
|  | R: 5' ATCTAAACAAACTTGGCGTGG 3' |  |  |
| *ccbe1* (zebrafish) | F: 5' AATCGCAACGACGAAGTACC 3' | NM_001163923.1 |  |
|  | R: 5' CCGGCACACACATCATAATC 3' |  |  |
| *e2f7* (zebrafish) | F: 5' CAAATCAGCAACTCCGGCAC 3' | NM_001045147.2 |  |
|  | R: 5' GCACTGTTGTTCCCGCTTTT 3' |  |  |
| *e2f8* (zebrafish) | F: 5' GCAGACGGTCGACCTAACAA 3' | NM_001347691.1 |  |
|  | R: 5' AGAAGTGGGATGATCGGGGA 3' |  |  |
| *E2F7* (human) | F: 5' GCCTGAAGCCAGCACTCCT 3' | NM_203394.3 |  |
|  | R: 5' CCCTCTCTGACCCTGACCCT 3' |  |  |
| *E2F8* (human) | F: 5' TTTGTGATGCTGTTTTTGGTGT 3' | NM_024680.4 |  |
|  | R: 5' GTATTTGGACTGATTTCTGGGC 3' |  |  |
| *CCBE1* (human) | F: 5' CAGGAGCAAGAGACCAGAAGG 3' | NM_133459.4 |  |
|  | R: 5' GAGGCAAAAAAGCCAAAAAGA 3' |  |  |
| *VEGFC* (human) | F: 5' CCTCTCCAAAAAGCTACACCG 3' | NM_005429.5 |  |
|  | R: 5' AAAAGCCTCACAGGAAACCG 3' |  |  |
| *FLT4* (human) | F: 5' GAGACAAGGACAGCGAGGAC 3' | U43143.1 |  |
|  | R: 5' CTGTGTCGTTGGCATGTACC 3' |  |  |
| *β-actin* (zebrafish) | F: 5' CGAGCAGGAGATGGGAACC 3' |  |  |
|  | R: 5' CAACGGAAACGCTCATTGC 3' |  |  |
| *β-actin* (human) | F: 5' AGCGAGCATCCCCCAAAGTT 3' |  |  |
|  | R: 5' GGGCACGAAGGCTCATCATT 3' |  |  |

| **Table S3. Sequences of primers used for amplifying probes for WISH** | | |  |
| --- | --- | --- | --- |
| gene | Primer sequence(5'to3') | GenBank ID |  |
|  |  |  |  |
| *ccbe1* | F: 5' CAGAGAAGTGTGTTCAGAAAGCA 3' | NM_001163923.1 |  |
|  | R: 5' GCTAATACGACTCACTATAGGGATACCCGT  CATAGCAGGTGC 3' |  |  |

| **Table S4. Sequences of primers for mRNA synthesis** | |
| --- | --- |
| gene | Primer sequence(5'to3') |
| *e2f7*-mRNA-F | 5' TAATACGACTCACTATAGGGC  AGCCTTGACACAGGATTTATTT 3' |
| *e2f7*-mRNA-R | 5' TGCTCTTCGTCTTTGGCACTT 3' |
| *e2f8*-mRNA-F | 5' TAATACGACTCACTATAGGGTT  TACCTCTGGGGTGCTTTTAC 3' |
| *e2f8*-mRNA-R | 5' CCGAATTGTGCTTTTGTTTTGT 3' |
| *ccbe1*-mRNA-F | 5' TAATACGACTCACTATAGGGCT  GAACTTCAAGACTGGATT 3' |
| *ccbe1*-mRNA-R | 5' CAGTTAAATAGCAGTCATTCTG 3' |

| **Table S5. Sequences of primers used for promoter methylation level assays** | | |  |
| --- | --- | --- | --- |
| gene | Primer sequence(5'to3') | Product length(bp) |  |
|  |  |  |  |
| *ccbe1* (zebrafish) | F(outter): 5' TTGGTTTTGTGGGTTTTTAGAGGTT 3' | 605 |  |
|  | R(outter): 5' AATCATTTTAAAAAATACTTAAAAAAAA 3' |  |  |
|  | F(inner): 5' TATTAGTGATGGGGTGTTTAGTTTTG 3' | 377 |  |
|  | R(inner): 5' ATTAACACAATCTCCTCACAACAAA 3' |  |  |
| *flt4* (zebrafish) | F(outter): 5' ATTAGAGAAAATTTAAGTTAATAGGGAGA 3' | 641 |  |
|  | R(outter): 5' CCCTCTAAAACCTACAAAATATCTACA 3' |  |  |
|  | F(inner): 5' GTTAATTGAGTTAGTCGGGGTTTA 3' | 492 |  |
|  | R(inner): 5' AACCTAAAACTCAACTCTCTTTCACT 3' |  |  |
| *CCBE1* (human) | F(outter): 5' AAAAAAAGTTAATGTGTATGTGATATGTG 3' | 827 |  |
|  | R(outter): 5' AATAAACTTCCTTCCTATTATCCATAA 3' |  |  |
|  | F(inner): 5' GAAAAGGTTGAGAAAATTTTTAAAG 3' | 273 |  |
|  | R(inner): 5' AAACCTTACATCTCCTTATCCTAAAC 3' |  |  |
| *FLT4* (human) | F(outter): 5' GGTTTGGAGAATGGAAATATAGGT 3' | 472 |  |
|  | R(outter): 5' AACAACCCTCTTACAAACTTCCGT 3' |  |  |
|  | F(inner): 5' TTTCCATAAGTGTGTTACATCTCATAA 3' | 368 |  |
|  | R(inner): 5' TCAGTTTGTCTCTAGAATTTTCCTGT 3' |  |  |

| **Table S6. Sequences of primers used for promoter activity assays** | |
| --- | --- |
| pCS2-2037/+403 *ccbe1*-EcoRI-F (zebrafish) | 5' GGAATTCTAGGCTATCA  GTGATGGGGTG 3' |
| pCS2-2037/+403 *ccbe1*-XbaI-R (zebrafish) | 5' GTCTAGACTTCGTCGTT  GCGATTTTG 3' |
| pGL3-2037/+403 *ccbe1*-KpnI-F (zebrafish) | 5' GGGTACCTAGGCTATCA  GTGATGGGGTG 3' |
| pGL3-2037/+403 *ccbe1*-XhoI-R (zebrafish) | 5' CCTCGAGCTTCGTCGTT  GCGATTTTG 3' |
| pCS2-2123/+150 *flt4*-EcoRI-F (zebrafish) | 5' GGAATTCAACAATAAC  CATCCTTGTCTACCA 3' |
| pCS2-2123/+150 *flt4*-XbaI-R (zebrafish) | 5' GTCTAGATCCTCTGACT  CACTCATCTGCC 3' |
| pGL3-2123/+150 *flt4*-KpnI-F (zebrafish) | 5' GGGTACCAACAATAAC  CATCCTTGTCTACCA 3' |
| pGL3-2123/+150 *flt4*-XhoI-R (zebrafish) | 5' CCTCGAGTCCTCTGACT  CACTCATCTGCC 3' |
| pCS2-131/+21 *CCBE1*-EcoRI-F (human) | 5' GGAATTCCCCTCCTCCG  TTTTCTTGTT 3' |
| pCS2-131/+21 *CCBE1*-XbaI-R ((human) | 5' GTCTAGATTGTCCTGAG  CGGCTTTAAT 3' |
| pGL3-131/+21 *CCBE1*-KpnI-F ((human) | 5' GGGTACCCCCTCCTCCG  TTTTCTTGTT 3' |
| pGL3-131/+21 *CCBE1*-XhoI-R ((human) | 5' CCTCGAGTTGTCCTGAG  CGGCTTTAAT 3' |
| pCS2-323/+238 *FLT4*-EcoRI-F ((human) | 5' GGAATTCTTGGAGAGA  GCTGGTAGTGG 3' |
| pCS2-323/+238 *FLT4*-XbaI-R ((human) | 5' GTCTAGACCTGTAATCC  CAGCTTCTCG 3' |
| pGL3-323/+238 *FLT4*-KpnI-F ((human) | 5' GGGTACCTTGGAGAGAG  CTGGTAGTGG 3' |
| pGL3-323/+238 *FLT4*-XhoI-R (human) | 5' CCTCGAGCCTGTAATCCC  AGCTTCTCG 3' |

| **Table S7. Sequences of primers used for ChIP-qPCR** | |
| --- | --- |
| *CCBE1*-ChIP-qPCR-F | 5' CCCTCCTCCGTTTTCTTGTT 3' |
| *CCBE1*-ChIP-qPCR-R | 5' TTGTCCTGAGCGGCTTTAAT 3' |
| *FLT4*-ChIP-qPCR-F | 5' TCTCAACCTCCTTCCCTGCT 3' |
| *FLT4-*ChIP-qPCR-R | 5' AGCAGGGAAGGAGGTTGAGA 3' |
